# Toll-like receptor 5 deficiency diminishes doxorubicin-induced acute cardiotoxicity in mice

**DOI:** 10.7150/thno.47516

**Published:** 2020-09-02

**Authors:** Zhen-Guo Ma, Chun-Yan Kong, Hai-Ming Wu, Peng Song, Xin Zhang, Yu-Pei Yuan, Wei Deng, Qi-Zhu Tang

**Affiliations:** 1 Department of Cardiology, Renmin Hospital of Wuhan University, Wuhan 430060, RP China.; 2 Hubei Key Laboratory of Metabolic and Chronic Diseases, Wuhan 430060, RP China.

**Keywords:** Apoptosis, cardiotoxicity, doxorubicin, NOX2, TLR5

## Abstract

**Rationale:** Clinical application of doxorubicin (DOX) is limited by its toxic cardiovascular side effects. Our previous study found that toll-like receptor (TLR) 5 deficiency attenuated cardiac fibrosis in mice. However, the role of TLR5 in DOX-induced cardiotoxicity remains unclear.

**Methods:** To further investigate this, TLR5-deficient mice were subjected to a single intraperitoneal injection of DOX to mimic an acute model.

**Results:** Here, we reported that TLR5 expression was markedly increased in response to DOX injection. Moreover, TLR5 deficiency exerted potent protective effects against DOX-related cardiac injury, whereas activation of TLR5 by flagellin exacerbated DOX injection-induced cardiotoxicity. Mechanistically, the effects of TLR5 were largely attributed to direct interaction with spleen tyrosine kinase to activate NADPH oxidase (NOX) 2, increasing the production of superoxide and subsequent activation of p38. The toxic effects of TLR5 activation in DOX-related acute cardiac injury were abolished by NOX2 deficiency in mice. Our further study showed that neutralizing antibody-mediated TLR5 depletion also attenuated DOX-induced acute cardiotoxicity.

**Conclusion:** These findings suggest that TLR5 deficiency attenuates DOX-induced cardiotoxicity in mice, and targeting TLR5 may provide feasible therapies for DOX-induced acute cardiotoxicity.

## Introduction

Doxorubicin (DOX), an anthracycline antibiotic, is a widely utilized and highly effective chemotherapeutic agent. DOX has been used for the treatment of a variety of cancers, including lymphoma, leukemia, breast cancer, and lung cancer 1. However, its clinical application is limited due to its toxic effects on the heart 2. Clinical manifestations of DOX-induced cardiotoxicity can be acute cardiotoxicity and chronic cardiomyopathy. DOX-induced acute cardiotoxicity occurred in 11% of patients, whereas the chronic cardiomyopathy of DOX occurred only in 1.7% of the patients undergoing DOX therapy 3-5. Characteristic change of acute cardiotoxicity caused by DOX included myofibrillar disruption, cardiomyocytes atrophy and vacuolated preapoptotic cells 6, while cumulative chronic cardiotoxicity could lead to ventricular dilation and congestive heart failure 7. Thus, effective therapeutic approaches for patients with DOX-induced acute cardiotoxicity are urgently needed.

The mechanism of DOX-induced acute cardiotoxicity involves increased reactive oxygen species (ROS), matrix metalloproteinase activation, and accumulation of cardiac inflammation, which eventually results in cardiomyocyte loss 8. A previous study found that DOX treatment impaired mitochondrial function, resulting in generation of free radicals and ROS in the myocardium 9,10. The NADPH oxidase (NOX) complex is one of the major ROS sources in the heart, and the primary isoforms expressed in the heart are NOX2 and NOX4 11,12. NOX isozymes are also implicated in DOX-induced cardiac injury 13. Zhao *et al*. found that NOX2 activation promoted cardiac injury in response to DOX chemotherapy in mice 14.

Recent studies have demonstrated an association between toll like receptors (TLRs) and NOX isozyme activation in host defense, suggesting that NOX activation is indispensable to TLR-mediated responses 15,16. TLRs are a subfamily of pattern recognizing receptors that serve as major regulators of innate and adaptive immunity 17. As a member of this family, TLR5 recognizes several pathogen-associated molecular patterns and danger-associated molecular patterns, leading to the release of inflammatory cytokines and pro-fibrotic mediators in myocardial infarction 18. Pharmacological activation of TLR5 by flagellin (FL) triggers acute cardiac contractile dysfunction in mice 19. Our previous study found that TLR5 deficiency attenuated cardiac fibrosis and dysfunction by inhibiting the endothelial-mesenchymal transition 20. A bioinformatics study revealed that TLR5 might be a candidate indicator of DOX-induced heart failure 21. Given these findings, we hypothesized that TLR5 deficiency would protect against DOX-induced cardiotoxicity in mice. This is the first report to clearly describe the role of TLR5 in DOX-induced cardiotoxicity.

## Materials and Methods

### Reagents

DOX (D1515, purity ≥98%) and FL (SRP8029, purity ≥95%) were purchased from Sigma-Aldrich (St. Louis, MO, USA). Small interfering RNAs targeting NOXs (siNOXs) were designed by RIBOBIO (Guangzhou, China). The adeno-associated virus 9 (AAV9) system carrying small hairpin RNA (shRNA) against NOX2 (AAV9-shNOX2) under the cTnT promoter or a negative control AAV9-shRNA was constructed by DesignGene Biotechnology (Shanghai, China). TLR5 neutralizing antibody was obtained from Invivogen (France). The selective NOX2 inhibitor (GSK2795039, HY-18950) was purchased from MedChemExpress (Shanghai, China). Primary antibodies were TLR5 (1: 200 dilution, #sc-30003, Santa Cruz Biotechnology), NOX1 (1: 200 dilution, #ab55831, Abcam), NOX2/gp91phox (1: 1000 dilution, #ab129068, Abcam), NOX3 (1: 1000 dilution, #PA5-39279, Invitrogen), NOX4 (1: 1000 dilution, #ab109225, Abcam), Bax (1: 1000 dilution, #2772, Cell Signaling Technology), Bcl-2 (1: 1000 dilution, #2870, Cell Signaling Technology), spleen tyrosine kinase (Syk, 1: 1000 dilution, #80460, Cell Signaling Technology), phospho-Syk (1: 1000 dilution, #2710, Cell Signaling Technology), phospholipase C-gamma1 (PLCγ1, 1:1000 dilution, #ab76155, Abcam), p-PLCγ1 (1: 1000 dilution, #ab76031, Abcam), protein kinase Cα (PKCα, 1: 1000 dilution, #ab32376, Abcam), HA-Tag (1: 1000 dilution, #ab9110, Abcam), GST (1: 1000 dilution, ab19256, Abcam), and glyceraldehyde 3-phosphate dehydrogenase (GAPDH, catalogue #2118, Cell Signaling Technology). 2′,7′-dichlorodihydrofluorescein diacetate (DCF-DA) was obtained from Invitrogen.

### Animal treatment protocol

All studies were approved by the Animal Care and Use Committee of Renmin Hospital of Wuhan University. Global TLR5 knockout (KO) mice (C57BL/6 background) were purchased from Jackson Laboratory, and these mice were used in our previous study 20. Heterozygous TLR5 deficient mice were interbred to establish homozygous TLR5 knockout mice and their wild-type littermates. All experimental animals were housed in a 12-h light-dark cycle (7 a.m./7 p.m.). TLR5 deficient mice (8-10 weeks old; body weight: 23-28 g) and age-matched wild-type (WT) littermates were used in this study. To mimic acute cardiotoxicity, mice were subjected to a single intraperitoneal injection of DOX (15 mg/kg) or the same volume of normal saline (NS) as previously described 22. To restore TLR5 in cardiomyocytes, global KO mice were given a single intravenous injection of 1×10^11^ viral genome particles of AAV9-TLR5 or AAV9-green fluorescent protein (GFP). Four weeks after AAV9 injection, TLR5 protein expression was assessed, and DOX was injected. Male mice were injected with FL (1 μg/mouse, every other day) or the same volume of 0.1% DMSO twice (first administration: 1 day before DOX treatment; second administration: 1 day after DOX treatment) via the orbital venous plexus 23. We selected this dose of FL because this dose is pathophysiologically and clinically relevant. Circulating FL reaches several hundred μg/L after lethal bacterial infection 24. To verify the hypothesis that the role of TLR5 in DOX-related cardiac injury was mediated by NOX2, mice were subjected to a single intravenous injection of AAV9-shNOX2 or a scrambled control (AAV9-shRNA) at a dose of 1×10^11^ viral genome particles/mouse 22,25,26. This AAV9 system was generated by DesignGene Bioscience (Shanghai, China). Four weeks after AAV9 injection, NOX2 protein expression was assessed, and DOX was injected. To enhance the clinical significance of our study, mice were given a TLR5 neutralizing antibody (5 μg/mouse, dissolved into 100 μl saline) or isotype-matched IgG together at the same time of DOX injection. All mice were weighed daily and sacrificed 3 days after DOX injection.

To observe survival rates in DOX-treated mice, we performed independent experiments. Mice were subjected to a single intraperitoneal injection of DOX (15 mg/kg) followed by 7 days of observation.

### Echocardiographic assessment

Three days after DOX injection, transthoracic echocardiography was performed in mice under inhaled isoflurane (1-1.5% via nosecore) anesthesia using two-dimensional guided M-mode echocardiography (Esaote SpA, Genoa, Italy) equipped with a 10-MHz linear transducer. Body temperature of the mice was maintained using a heated imaging platform and warming lamps. Images of the left ventricle were acquired along with the long and short axes to assess left ventricular ejection fraction (EF) 25-28.

### Histological analysis and staining

Heart tissues were collected and fixed in 10% formalin, embedded in paraffin and sectioned. To assess cardiac fiber morphology, sections were stained with hematoxylin and eosin (H&E). To evaluate superoxide production in the myocardium, fluorescent dihydroethidium (DHE) was used. Briefly, fresh heart samples were sectioned and incubated with DHE (1 μM) for 30 min. The terminal deoxynucleotide transferase (TdT)-mediated dUTP nick end labeling (TUNEL) technique was used to detect apoptotic cell death using a commercially available kit (Millipore, USA). Images were captured using an Olympus DP72 microscope digital imaging system and were analyzed using the NIH ImageJ program.

### Western blot and quantitative real-time PCR

Heart tissues were homogenized in radioimmunoprecipitation assay buffer, and total proteins were extracted and normalized to the same level. Nuclear proteins were acquired using NE-PER™ Nuclear and Cytoplasmic Extraction Reagents (Thermo Scientific), and membrane proteins were acquired using Mem-PER™ Plus Membrane Protein Extraction Kit (Thermo Scientific). These proteins were subjected to SDS-PAGE and transferred onto PVDF membranes (#FL00010; EMD Millipore, MA, USA) 29. After blocking with 5% nonfat milk at room temperature for an hour, membranes were incubated overnight with specific antibodies at 4 ℃. Membranes were then probed with secondary antibodies at room temperature for one hour. Immunoblots were scanned and analyzed using the Odyssey Infrared Imaging System (LI-COR Biosciences, Lincoln, NE, USA).

For detection of mRNA expression, RNA extraction and reverse transcriptase-polymerase chain reaction (Transcriptor First Strand cDNA Synthesis Kit, Roche, Basel, Switzerland) were performed. The LightCycler 480 SYBR Green Master Mix (cat. No 4896866001; Roche) was used for quantification of real time PCR. The fold change in levels of target mRNAs between groups was correlated to levels of GAPDH. Data were analyzed using the 2-^ΔΔCT^ method.

### Assessment of serum biomarkers

Blood samples were obtained from the posterior orbital venous plexus in anaesthetized mice one day post DOX injection, and serum was separated for measurement of the lactate dehydrogenase (LDH) isoenzyme using a colorimetric LDH cytotoxicity assay (G1782, Promega) and creatine kinase isoenzyme-MB (CK-MB, catalogue #H197, Nanjing Jiancheng Bioengineering Institute, Nanjing, China) according to the kit instructions.

### Cell culture and treatment

Neonatal rat cardiomyocytes (NRCMs) were isolated according to our previous study 26. Briefly, hearts were digested in 0.25% trypsin at 37 ℃. Next, NRCMs were purified by removing other heart cell populations via a differential attachment technique. These cells were maintained in a humidified atmosphere with 5% CO_2_ at 37 °C and cultured in Dulbecco's Modified Eagle's Medium (DMEM, GIBCO, C11995), containing 10% fetal bovine serum (FBS, GIBCO, 10099). To deplete TLR5 expression, siRNAs were utilized to knock down expression of TLR5. The small interfering RNA targeting (siTLR5) target sequence is 5'-GGACTGCGATGAAGAGGAA-3'. A scrambled siRNA was used as a control. Cells were transfected with siRNA or siTLR5 (50 nM) using Lipofectamine TM 6000 for 4 h. Then, the media was replaced with DMEM containing 10% FBS, and cells were subjected to DOX (1 μM) or PBS for 24 h 30. Cells were also treated with FL (50 ng/mL dissolved in 0.1% DMSO) or vehicle (0.1% DMSO) for 24 h 19. To identify specific species of ROS, we detected the production of hydrogen peroxide and superoxide using a hydrogen peroxide assay kit (Biovision, #K265-200) and Superoxide Anion Assay Kit (Sigma-Aldrich, # CS1000). To detect nuclear factor-κB (NF-κB) transcriptional activation, we used the TransAM® NF-κB activation assay kit (Active Motif, USA). To confirm that alteration of inflammation was secondary to overproduction of ROS in DOX+FL-treated cells, N-acetyl-L-cysteine (NAC, #A7250, Sigma-Aldrich) or the NADPH oxidase inhibitors diphenylene iodonium (DPI, #D2926, Sigma-Aldrich) or apocynin (#178385, Sigma-Aldrich) were used. To test the hypothesis that TLR5 exerts its function via NOX2, cells were transfected with several siRNAs targeting NOX isoenzymes (50 nM) using Lipofectamine TM 6000 for 4 h. These siRNAs were generated by RiboBio Co. Ltd. (Guangzhou, Guangdong, China). NRCMs were also pretreated with a NOX2 inhibitor (GSK2795039, 25 μM, MedChemExpress) for 24 h. To confirm the role of Syk, NRCMs were pretreated with piceatannol (100 μM), a pharmacological inhibitor of Syk, for 24 h followed by DOX treatment. Cells were transfected with siSyk (50 nM) or siRNA using Lipofectamine TM 6000 for 4 h. To inhibit p38 activity, NRCMs were treated with SB203580 (10 μM, Selleck) for 24 h. Cell viability was assessed using the Cell Counting Kit (catalogue #C0038, Beyotime) according to the manufacturer's instructions.

### Immunoprecipitation and GST pull down assay

Hearts were lysed and incubated with antibodies targeting TLR5 or IgG at 4 °C overnight. Subsequently, lysates were collected with Protein A+G Agarose beads (Calbiochem #IP05). These beads were washed with immunoprecipitation buffer, and protein complexes were released and separated by SDS-PAGE.

HEK293T cells were transfected with a HA-Syk plasmid for 48 h. Next, cells were lysed and HA-Syk was prepared according the IP assay. Rosetta (DE3) *Escherichia coli* cells were transformed with the pGEX-6p-1-GST-TLR5 vector and then induced by IPGT (0.5 mM, Thermo Fisher Scientific). Next, extracts were reacted with glutathione-sepharose 4B beads (#17075601, GE Healthcare) at 4 °C for 60 min. Bead complexes were reacted with immunopurified HA-Syk at 4 °C for 6 h. After reaction, these complexes were eluted in elution buffer and separated by SDS-PAGE.

### Measurement of lipid peroxidation and NADPH activity

To evaluate levels of lipid peroxidation, malondialdehyde (MDA) and 4-hydroxynonenal (4-HNE) were detected using an MDA assay kit (#A003-1-2, Nanjing Jiancheng Bioengineering Institute) and 4-HNE assay kit (ab238538, Abcam). Assays were performed according to the manufacturer's instructions.

NADPH oxidase activity in hearts was detected using an NADPH oxidase assay kit (#A127-1-1) obtained from the Nanjing Jiancheng Bioengineering Institute.

### Measurement of redox status

NRCM cells were plated in 6-well culture plates for 48 h followed by treatment with DOX (1 μM) for 24 h. Cells were then incubated with DCF-DA (10 μM) at 37 °C for 30 min, and then samples were analyzed by fluorescence microscopy (Olympus, Tokyo, Japan).

### Statistical analysis

Data in our study were analyzed using SPSS (IBM, Chicago). All data are presented as the mean±standard error (SEM). A value of *P* ≤0.05 was considered significant. Differences between groups were evaluated using two-tailed Student's t-tests to compare two groups and one-way analysis of variance (ANOVA) to compare three or more groups followed by Tukey post hoc test. A repeated-measures ANOVA was also used to evaluate body weight gain and cell viability. Survival data were assessed by the Kaplan-Meier method, and survival curves were compared using the Mantel-Cox log-rank test.

## Results

### TLR5 deficiency attenuates DOX-induced cardiac injury and improves cardiac function in mice

**Figure 1:**
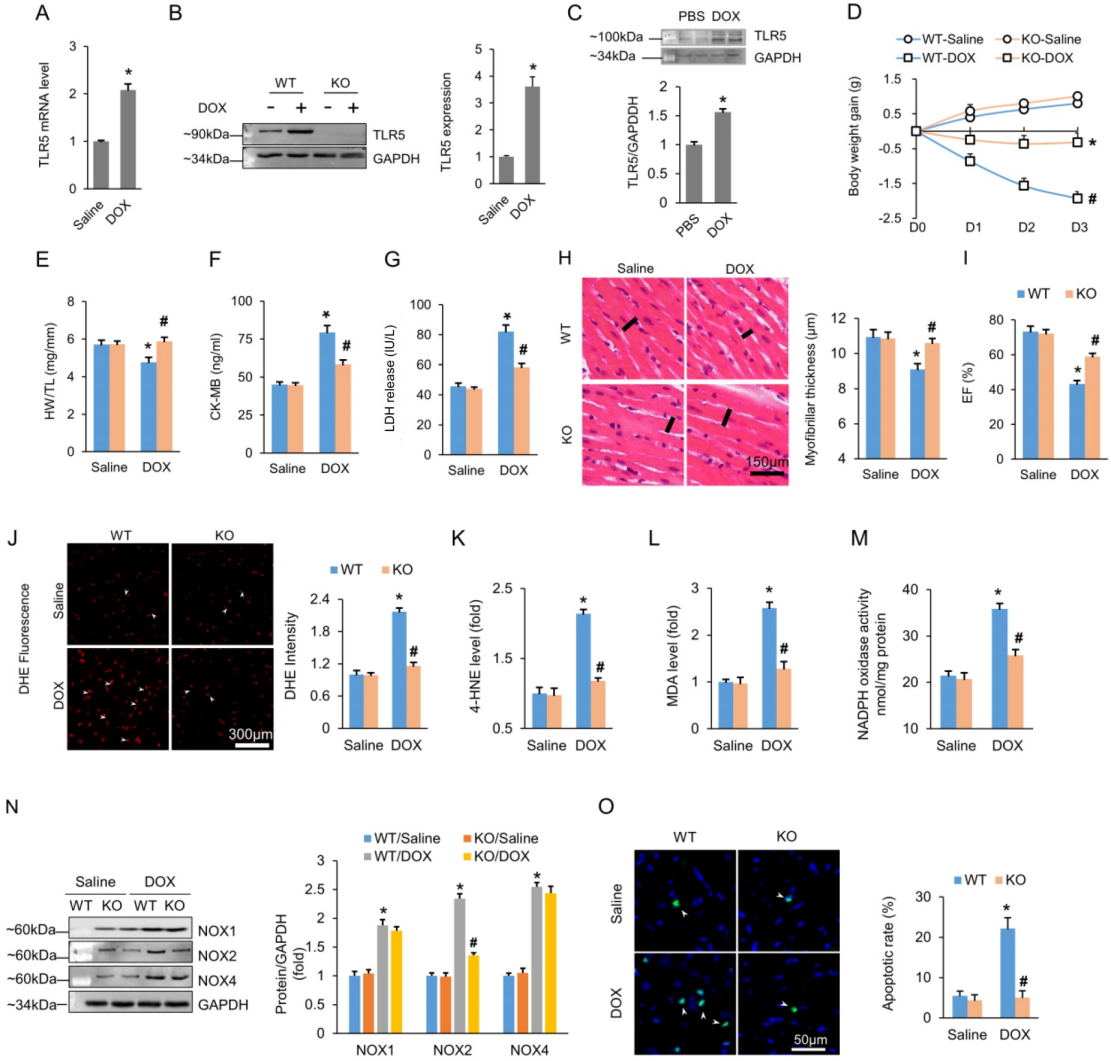
** TLR5 deficiency attenuates the DOX-induced cardiotoxic effects in mice.** (A-B) TLR5 mRNA and protein expression in the hearts at three days after DOX injection (n = 6-8). (C) Expression of TLR5 protein in NRCMs received DOX for 24 h (n = 6). (D) Body weight change (n = 8). (E) The ratio of heart weight to tibia length (n = 8). (F-G) Serum concentration of CK-MB and LDH (n = 6). (H) H&E staining. Black line indicates the thickness of the myofibril (n = 8). (I) Ejection fraction in TLR5 KO or WT mice with or without DOX (n = 6). (J) DHE staining (n = 6). (K-L) The levels of 4-HNE and MDA in DOX-treated hearts (n = 6). (M) NADPH oxidase activity (n = 6). (N) Protein expression of NOX isoenzymes (n = 6). (O) Apoptosis measured by TUNEL staining (n = 6). ^*^P < 0.05 versus WT Saline; ^#^*P <* 0.05 versus WT DOX group.

To investigate the role of TLR5 in DOX-induced cardiomyopathy, we initially assessed expression of TLR5 in ventricular samples of mice. We found that TLR5 mRNA and protein levels were significantly increased in the hearts three days after DOX injection (Figure 1A-B). In addition, TLR5 upregulation was observed in isolated neonatal rat cardiomyocytes that were stimulated with DOX for 24 h compared to PBS-treated controls (Figure 1C). The elevation in TLR5 expression induced by DOX prompted us to investigate whether TLR5 plays a role in DOX-induced cardiotoxicity. We then utilized global TLR5 knockout (KO) mice and age-matched wild type (WT) littermates to explore the role of TLR5 protein in DOX-induced cardiomyopathy in a 3-day short-term trial (Figure S1A). Results showed that body weight loss induced by DOX injection was attenuated in TLR5 KO mice (Figure 1D). In response to DOX injection, the ratio of heart weight to tibia length (HW/TL) was significantly increased in TLR5 deficient mice compared to heart samples from WT littermate controls (Figure 1E). Next, we detected CK-MB and LDH isoenzyme to assess myocardial injury. As shown in Figure 1F-G, the increased levels of CK-MB and LDH were reduced in response to TLR5 deficiency. Characteristic pathological change caused by DOX included myofibrillar disruption 31. In our study, histological analysis revealed that DOX-induced loss of myofibrillar thickness was largely attenuated in TLR5 KO mice (Figure 1H). Next, cardiac function was assessed by echocardiography, and we found that TLR5 deficiency largely improved EF after DOX injection (Figure 1I). TLR5 depletion did not impact heart rate, ventricle chamber dimensions or systolic blood pressure (Figure S1B-D).

Next, we examined myocardial inflammation and found that mRNA levels of tumor necrosis factor-α (TNF-α) and interleukin-1β (IL-1β) reached their peak on the first day, subsequently decreasing on the third day post DOX injection (Figure S1E). TLR5 deficiency attenuated myocardial TNF-α and IL-1β mRNA levels after DOX injection (Figure S1E). DOX-induced nuclear translocation of NF-κB was also inhibited by TLR5 deficiency (Figure S1F). Further experiments were performed to evaluate the impact of TLR5 on superoxide generation. Immunofluorescence staining showed that TLR5 deficiency markedly reduced intracellular superoxide production after DOX injection (Figure 1J). TLR5 deficiency also reduced levels of 4-HNE and MDA in DOX-treated hearts (Figure 1K-L). The activity of NAPDH oxidase was also increased in the hearts of DOX-treated control mice, and these effects were blocked by TLR5 deficiency (Figure 1 M). Next, we detected expression of NOX isozymes in both WT and TLR5 KO mice. As shown in Figure 1N, the increased NOX2 protein expression in DOX-treated hearts was reduced by TLR5 deficiency. However, TLR5 deficiency had no obvious effect on expression of NOX1 or NOX4 induced by DOX.

Cardiac apoptosis contributes to DOX-related cardiomyopathy 32. The apoptotic rate was higher in DOX-treated animals compared to animals injected with saline. However, TLR5 KO mice injected with DOX maintained an apoptotic rate close to that of control animals (Figure 1O). Further analyses revealed that TLR5 deficiency also suppressed protein expression of the pro-apoptotic Bax gene but upregulated protein expression of Bcl-2 (an anti-apoptotic gene) 3 days after DOX (Figure S1G).

**Figure 2:**
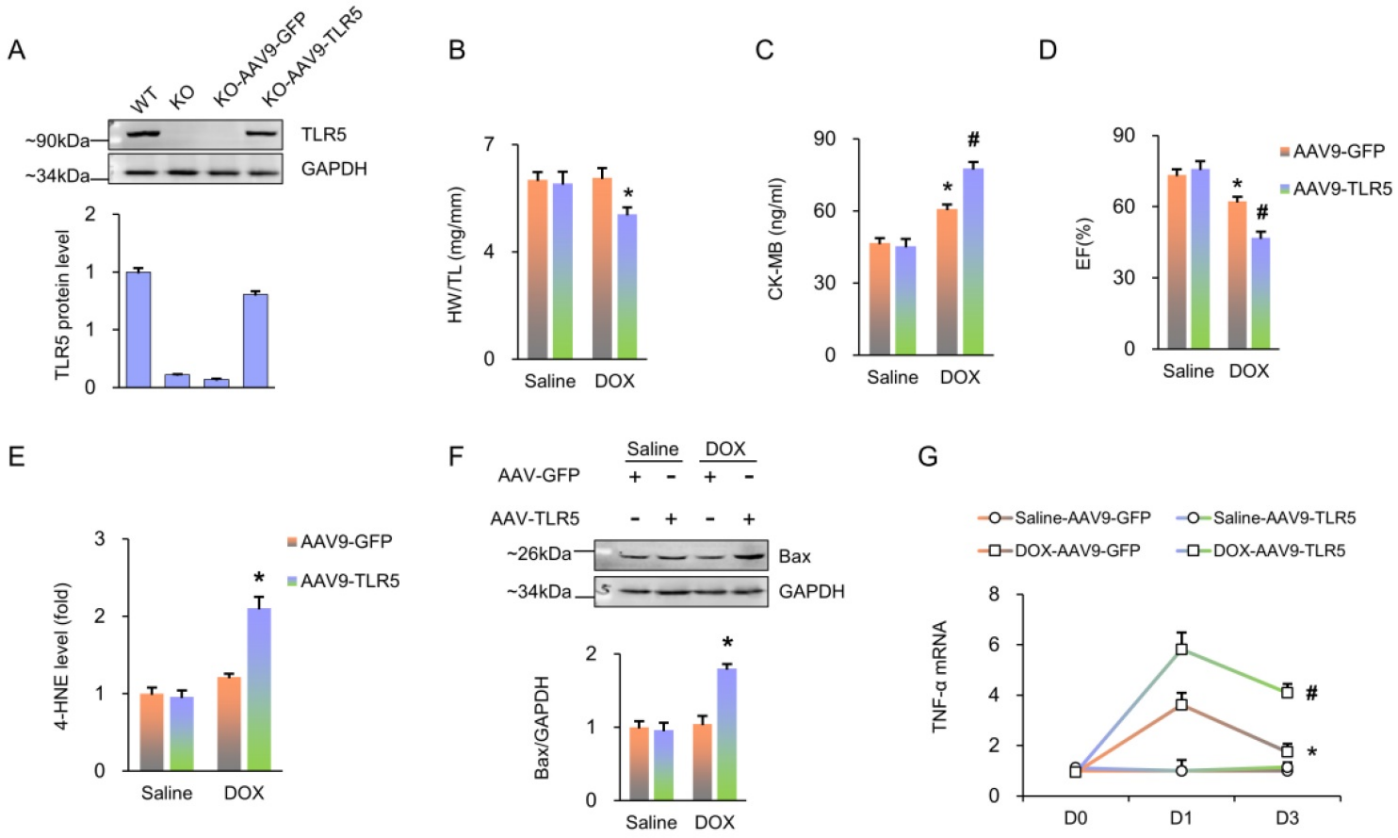
** TLR5 restoration promotes DOX-induced damage in TLR5-deficient mice**. (A) TLR5 protein expression (n = 6). (B) The ratio of heart weight to tibia length (n = 10). (C) Serum concentration of CK-MB (n = 6). (D) Ejection fraction in mice with AAV9-TLR5 (n = 6). (E) The levels of 4-HNE in DOX-treated hearts (n = 6). (F) Bax expression (n = 6). (G) The mRNA levels of inflammatory factors (n = 6). ^*^P < 0.05 versus KO Saline; ^#^*P <* 0.05 versus KO DOX group.

Next, we used a viral-mediated rescue approach to restore TLR5 expression in cardiomyocytes. Global KO mice were given a single injection of AAV9-TLR5. With this infection, TLR5 expression in KO mice reached 78.2% of that observed in WT mice treated with AAV9-GFP (Figure 2A). Of note, compared to the AAV9-GFP group, transduction with AAV9-TLR5 promoted DOX-related cardiac injury, as indicated by decreased HW/TL and EF, as well as increased levels of CK-MB, 4-HNE, Bax protein and TNF-α mRNA (Figure 2B-G).

### FL treatment aggravates cardiotoxicity induced by DOX *in vivo*

**Figure 3:**
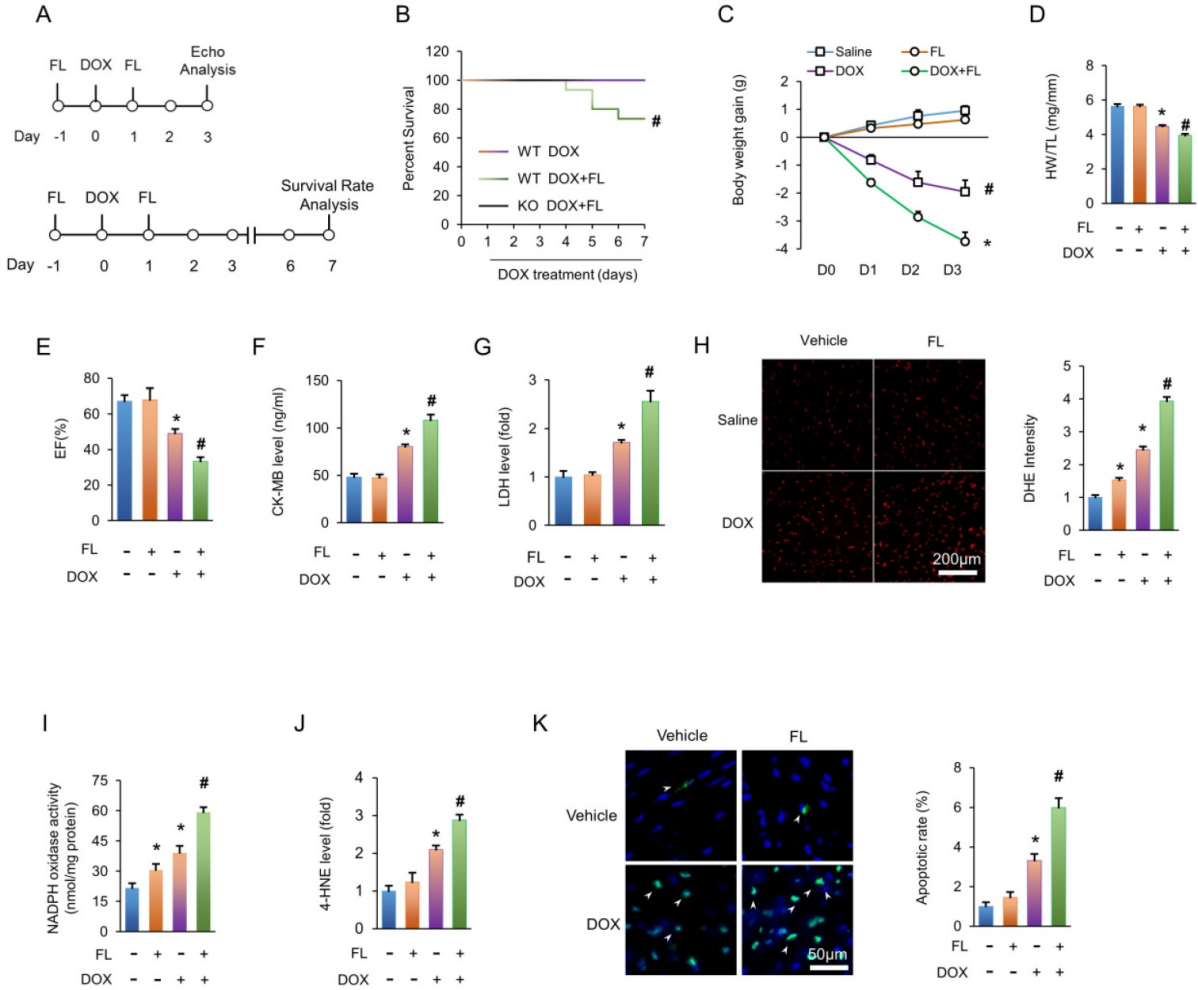
** FL treatment aggravates the DOX-induced cardiotoxic effects in mice.** (A) Experimental protocol. (B) Survival for FL+DOX-treated mice (n = 15). (C) Body weight change at three days after DOX injection (n = 12). (D) The ratio of heart weight to tibia length at three days after DOX injection (n = 12). (E) Ejection fraction at three days after DOX injection (n = 6). (F-G) CK-MB and LDH in DOX+FL-treated mice at three days after DOX injection (n = 6). (H) DHE staining at three days after DOX injection (n = 5). (I) NADPH oxidase activity at three days after DOX injection (n = 6). (J) The levels of 4-HNE in DOX-treated hearts at three days after DOX injection (n = 6). (K) TUNEL staining at three days after DOX injection (n = 6). *P < 0.05 versus NS group; ^#^*P <* 0.05 versus DOX group.

To determine the effects of TLR5 activation upon DOX-induced cardiotoxicity, FL was applied in WT littermate mice together with DOX (Figure 3A). Results revealed that DOX alone did not result in any animal death for 7 days after injection. Conversely, survival rates were significantly decreased in mice treated with DOX+FL, and the deleterious effect of FL was completely blocked in TLR5-deficient mice (Figure 3B). Under basal conditions, FL administration did not affect body weight of mice. However, FL induced a dramatic exacerbation in weight loss caused by DOX (Figure 3C). FL also exacerbated cardiotoxicity induced by DOX, as indicated by decreased HW/TL and ejection fraction (EF) and increased CK-MB and LDH (Figure 3D-G). DHE staining revealed that FL promoted the production of myocardial ROS in saline or DOX-treated hearts (Figure 3H). FL alone increased NADPH oxidase activity even without DOX injection (Figure 3I). Moreover, we found that FL further increased elevated NADPH oxidase activity in DOX-treated hearts (Figure 3I). FL alone increased mRNA levels of TNF-α and IL-1β, and further increased elevation of these inflammatory markers in DOX-treated hearts (Figure S2A-B). DOX-induced 4-HNE production was also enhanced by FL treatment (Figure 3J). In line with these data, we observed elevated rates of apoptosis in mice with DOX+FL compared to DOX treatment alone (Figure 3K).

### The aggravating effects of TLR5 in DOX-induced cardiomyocyte damage are mediated by NOX2

**Figure 4:**
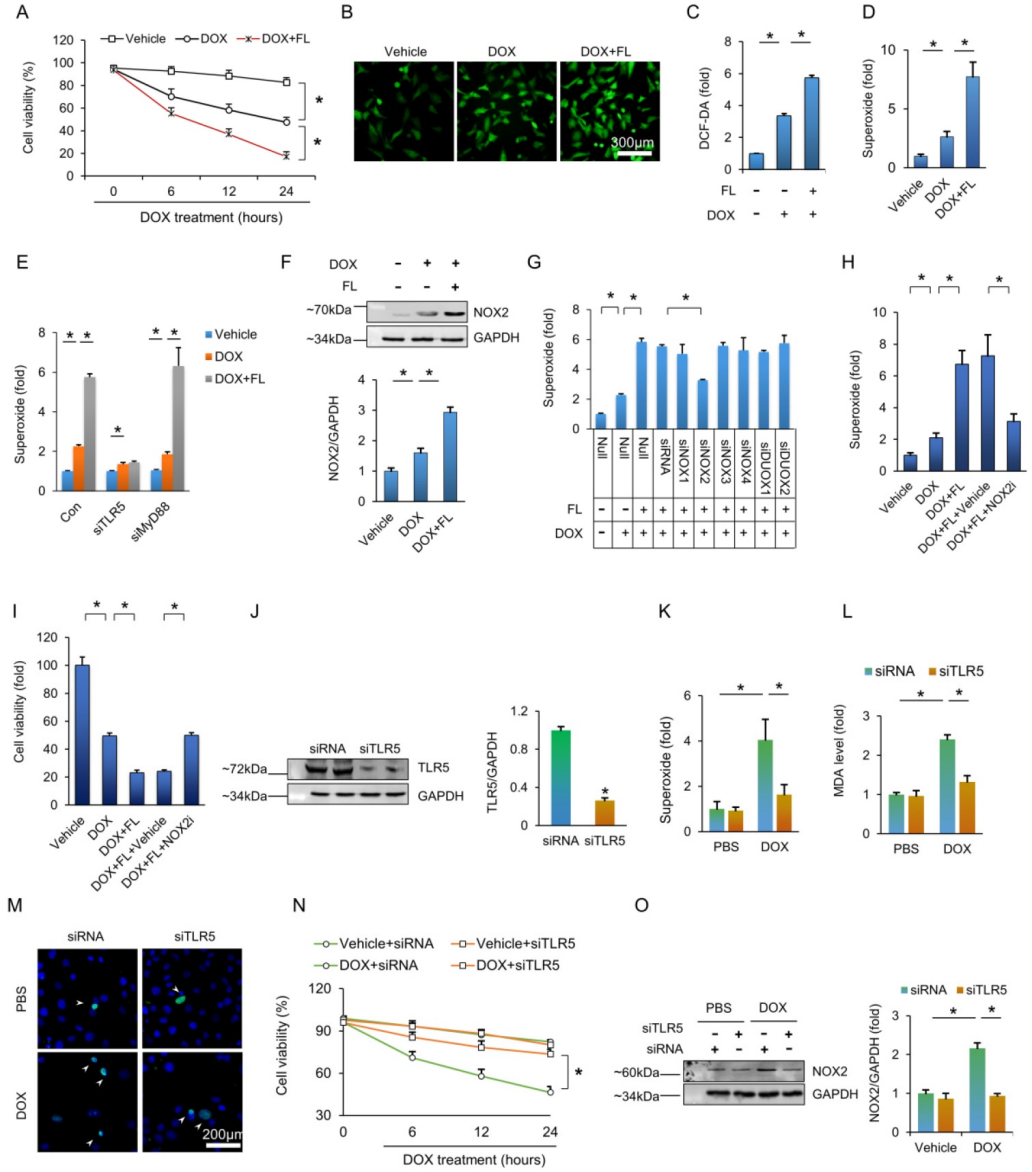
** TLR5 promotes DOX-induced toxic effects via activation of NOX2 *in vitro*.** (A) Cell viability after FL treatment (n = 6). (B) DCF-DA staining indicated the production of ROS. (C) ROS production as detected by ELISA (n = 6). (D-E) The production of superoxide (n = 6). (F) The NOX2 protein expression (n = 6). (G) The production of superoxide after knockdown of NOX isoenzymes (n = 6). (H) Superoxide production after NOX2 inhibition (n = 6). (I) Cell viability after NOX2 inhibition (n = 6). (J) TLR5 expression (n = 6). (K) The production of superoxide after TLR5 deficiency (n = 6). (L) MDA production after TLR5 deficiency (n = 6). (M) TUNEL staining. (N) Cell viability after TLR5 deficiency (n = 6). (O) NOX2 expression after TLR5 depletion (n = 6). *P < 0.05 versus matched control. The data are expressed as the mean ± SEM from six independent experiments.

Next, we used FL to stimulate NRCMs. FL treatment impaired cell viability under basal conditions (Figure S3A), and FL treatment further decreased cell viability after DOX exposure (Figure 4A). Exposure of NRCMs to DOX resulted in increased fluorescence intensity of DCF-DA, and this effect was largely promoted by FL treatment (Figure 4B-C). To identify the specific species of ROS generated, we assessed production of hydrogen peroxide and superoxide. FL treatment did not affect the production of hydrogen peroxide in DOX-treated cells (Figure S3B) but greatly increased production of superoxide in PBS and DOX-treated cells (Figure S3C, Figure 4D). Moreover, the aggravating effect of TLR5 in the production of superoxide in response to DOX exposure was prevented by TLR5 deficiency, not myeloid differentiation factor 88 (MyD88) deficiency, suggesting that this effect is TLR5-dependent rather than MyD88-dependent (Figure S3D, Figure 4E). Next, we examined expression of NOX2 protein in NRCMs. The results demonstrated that FL increased the expression of NOX2 and enhanced the protein expression of NOX2 in NRCMs with DOX stimulation (Figure S3E, Figure 4F). Moreover, the effects of FL on superoxide production in DOX-treated cells were completely blocked by siNOX2 but not siRNAs targeting other NOX isozymes (Figure 4G, Figure S4A-B). In line with this finding, the effects of FL on superoxide production and cell viability were offset by a NOX2 inhibitor (Figure 4H-I). We also detected NF-κB activation and found that FL alone activated NF-κB *in vitro* (Figure S5A). FL also further promoted NF-κB activation and increased TNF-α mRNA levels in DOX-treated NRCMs, and this pathological effect was blocked by a TLR5 neutralizing antibody, a nonspecific ROS scavenger N-acetyl-L-cysteine (NAC), or the NADPH oxidase inhibitors diphenylene iodonium (DPI) and apocynin (Figure S5B-C). Having demonstrated that TLR5 deficiency preserves cardiac function and attenuates oxidative damage in mice injected with DOX, we next assessed whether TLR5 deletion protected against DOX damage in NRCMs. As expected, TLR5 silencing decreased the production of superoxide in DOX-treated cells (Figure 4J-K). The elevated MDA content in DOX-treated NRCMs was suppressed by TLR5 deletion (Figure 4 L). TLR5 deficiency also decreased apoptotic cell death induced by DOX *in vitro* (Figure 4 M). The viability of DOX-treated NRCMs was markedly improved in response to TLR5 deficiency (Figure 4N). Subsequent detection also revealed that the increase of NOX2 in DOX-treated cells was remitted by transfection of siTLR5 (Figure 4O).

### NOX2 is responsible for the role of TLR5 in DOX-related cardiac injury

**Figure 5:**
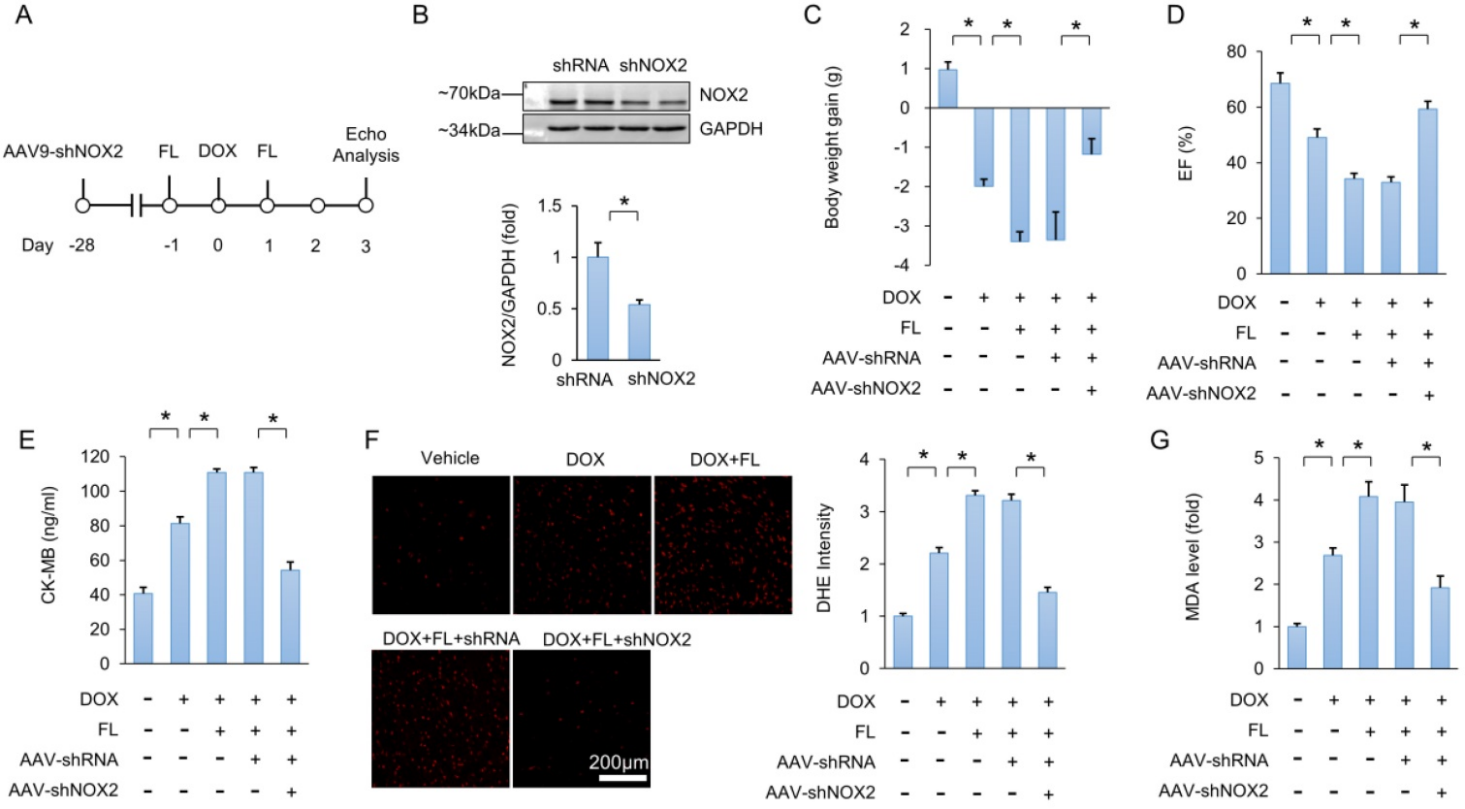
** NOX2 deficiency abolishes the toxic effects caused by FL in mice.** (A) Experimental protocol. (B) NOX2 protein expression (n = 6). (C) Body weight gain (n = 10). (D) EF in the indicated groups (n = 8). (E) CK-MB levels (n = 6). (F) DHE staining and statistical results (n = 6). (G) MDA level after NOX2 deficiency (n = 6). *P < 0.05 versus matched control.

Next, we utilized a viral-mediated approach to deplete myocardial NOX2. Mice were subjected to a single intravenous injection of AAV9-shNOX2 or a negative control AAV9-shRNA (Figure 5A). Four weeks after AAV9 injection, NOX2 protein expression was detected. Results showed that NOX2 protein expression was decreased in hearts injected with AAV9-shNOX2 compared to hearts that received AAV9-shRNA injection (Figure 5B). FL treatment aggravated DOX-induced cardiac injury, as indicated by decreased body weight, EF, and increased CK-MB (Figure 5C-E). However, this phenotype was largely prevented by NOX2 deficiency (Figure 5C-E). Increased DHE intensity and MDA levels in DOX+FL-treated mice were suppressed by AAV9-shNOX2 injection (Figure 5F-G). In addition, increased TNF-α and IL-1β mRNA levels in DOX+FL-treated mice were suppressed by AAV9-shNOX2 injection (Figure S6A-B).

### Spleen tyrosine kinase (Syk) is responsible for TLR5-mediated NOX2 activation

**Figure 6:**
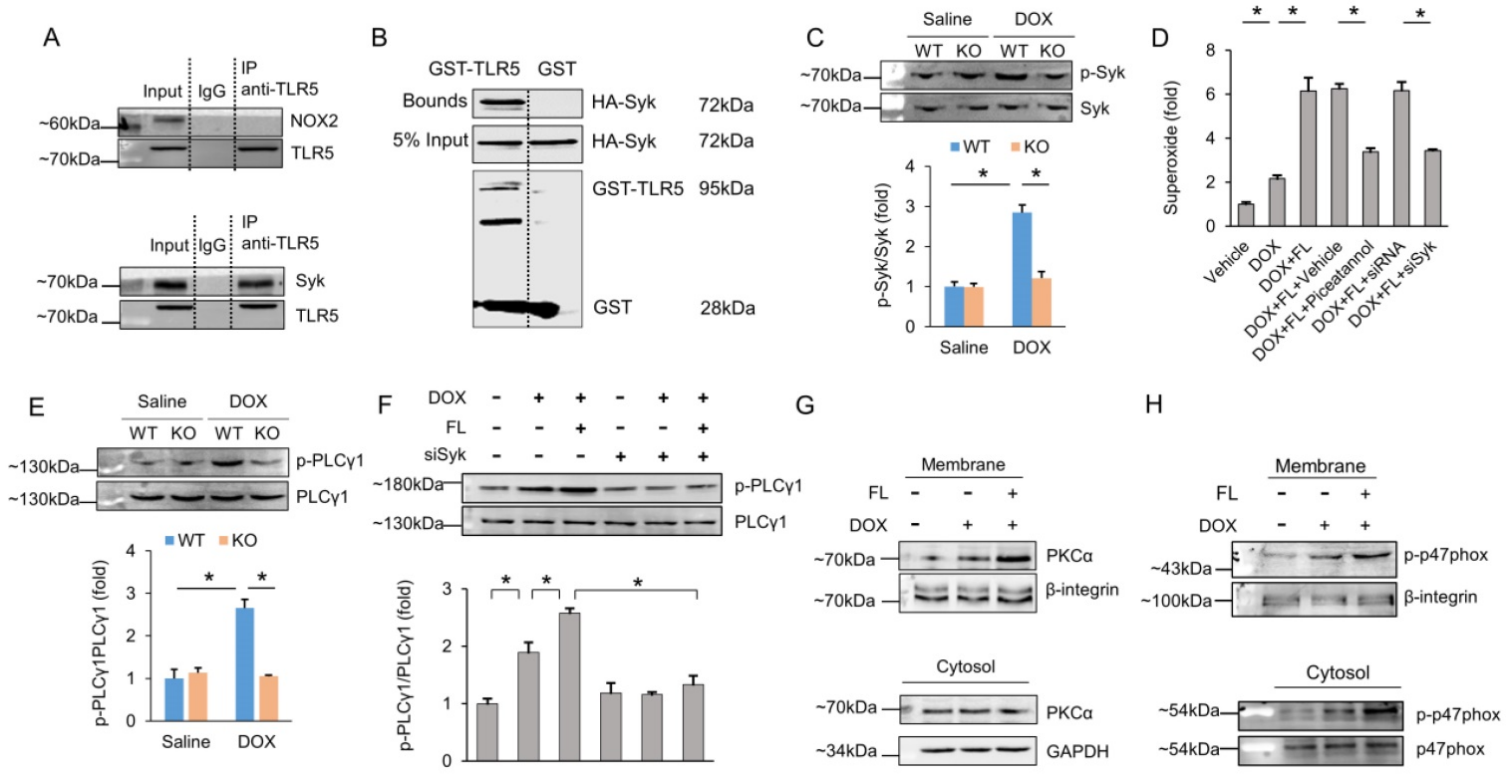
** TLR5 regulates NOX2 through direct physical interaction with Syk.** (A) The hearts were lysed and reacted with antibodies targeting TLR5, and then complexes were directly subjected to immunoblot analysis with NOX2 antibody or Syk antibody. (B) Immunoblot of a GST pull-down assay. (C) The p-Syk expression in TLR5-deficient mice (n = 6). (D) The production of superoxide after Syk inhibition (n = 6). (E-F) The PLCγ1 expression (n = 6). (G-H) DOX induced translocation of PKCα and p47phox from cytosol to the plasma membrane (n = 6). *P < 0.05 versus matched control. The data are expressed as the mean ± SEM from six independent experiments.

Next, we attempted to uncover the specific molecule that resulted in NOX2 activation after FL treatment. We first examined whether TLR5 and NOX2 interacted. The data in our study demonstrated that TLR5 does not interact with NOX2 (Figure 6A), implying that TLR5 does not directly activate NOX2. A canonical mechanism of NOX2 activation requires activated PLCγ1-dependent PKC activation 33. Syk phosphorylates PLCγ1 and plays a key role in the production of ROS 34,35. Given this, we assessed the interaction between Syk and TLR5. Surprisingly, our immunoprecipitation assay results revealed that Syk binds to TLR5 (Figure 6A). Next, we performed a GST pull-down assay with GST-tagged TLR5 to determine whether binding of TLR5 to Syk occurs via a direct interaction. Consistent with the immunoprecipitation results, Syk was eluted along with TLR5 (Figure 6B). In response to DOX, phosphorylation of Syk was also increased. However, this pathological elevation was largely prevented by TLR5 deficiency in mice (Figure 6C). Next, we examined whether Syk was responsible for NOX2 activation and superoxide generation. Pretreatment of NRCMs with piceatannol, a pharmacological inhibitor of Syk, significantly reduced superoxide production in DOX+FL-treated cells (Figure 6D). We also knocked down Syk to 31.8% of its WT levels in cells infected with siRNA and found that superoxide production in Syk-deficient cells was significantly decreased in response to FL+DOX (Figure 6D, Figure S7). Syk activation is known to phosphorylate PLCγ1, which attracts PKC to the membrane, leading to NOX2 activation and superoxide generation 35. In response to DOX, phosphorylation of PLCγ1 was increased, and this elevation was largely prevented by TLR5 deficiency in mice (Figure 6E). Moreover, FL further increased phosphorylation of PLCγ1 in DOX-treated cells, and this effect was prevented by Syk deficiency (Figure 6F). Phosphorylation of p47phox by PKC results in activation of NOX2 36, so we detected activation of PKCα and p47phox in terms of its membrane translocation. The data in our study indicated that DOX induced translocation of PKCα and p47phox from the cytosol to the plasma membrane, and this action was promoted by FL treatment (Figure 6G-H).

### ROS produced by NOX2 results in p38 activation

It has been reported that the generation of ROS results in activation of p38 37. Therefore, we first assessed alteration of p38 in TLR5-deficient mice. The activation of p38 induced by DOX injection was prevented by TLR5 deficiency (Figure S8A). FL alone induced activation of p38 (Figure S8B). In addition, FL increased phosphorylation of p38 in DOX-treated cells, and this effect was inhibited by NOX2 deficiency (Figure S8C). We also found that an inhibitor of p38 (SB203580) dramatically improved cell viability in DOX+FL-treated cells (Figure S8D).

### TLR5 neutralizing antibody abolishes cardiac damage caused by DOX in mice

To enhance the clinical significance of our study, mice were given a TLR5 neutralizing antibody or isotype-matched IgG together at the same time of DOX injection. TLR5 depletion significantly reduced DOX-related cardiac injury, as indicated by restored body weight, improved EF, and decreased CK-MB, 4-HNE, MDA and apoptosis in mice (Figure S9A-F). TLR5 depletion significantly decreased mRNA levels of TNF-α and IL-1β in DOX-treated hearts (Figure S9G).

## Discussion

Acute cardiotoxicity is more common than previously thought and predicts poor outcomes 38. It has been estimated that DOX-induced acute cardiotoxicity occurs within days in approximately 10% of patients subjected to DOX treatment 39. Here, we found that TLR5 deficiency attenuated myocardial oxidative damage, prevented apoptosis, and improved cardiac function in mice. Our results demonstrated that TLR5 interacts with Syk to activate NOX2, increasing production of superoxide and subsequent activation of p38. Importantly, the role of TLR5 in DOX-related cardiac injury was further validated using TLR5 neutralizing antibody, suggesting promising application of this neutralizing antibody for DOX-related acute cardiotoxicity.

Our previous study found that TLR5 deficiency prevented pressure overload-induced cardiac remodeling in mice 20. However, the functional relevance of TLR5 in DOX-related acute cardiotoxicity was unknown. Here, we observed increased TLR5 expression in DOX-stimulated cardiomyocytes and rodent hearts. Deficiency of TLR5 dramatically attenuated DOX-induced acute cardiotoxicity and cardiac function. However, TLR5 deficiency exacerbated cardiac injury induced by ischemia-reperfusion in mice 40. This discordance might be explained by the different roles of TLR5 in different disease models.

Previous studies suggest a potential role for TLR5 in inflammatory diseases 41,42. One of the landmarks of DOX-related acute cardiotoxicity is cardiac inflammation 43. Therefore, we detected myocardial inflammation in TLR5-deficient mice in response to DOX treatment. TLR5 deficiency decreased myocardial inflammatory factors and inhibited nuclear translocation of NF-κB at three days after DOX injection. However, a study reported that DOX treatment didn't cause a significant alteration of NF-κB binding activity at five days^44^. Furthermore, we found that the inflammatory response caused by a TLR5 agonist was ROS-dependent. These findings suggested that inflammatory response was not the main biological factors and secondary to ROS production during TLR5-mediated acute DOX injury. DOX has been postulated to induce cardiotoxicity through ROS generation 45. Excessive generation of electrophiles and oxidants after DOX treatment results in oxidation of membrane lipids and production of highly reactive 4-HNE, impairing cardiac function 46. The data in our study demonstrated that TLR5 deficiency markedly reduced myocardial superoxide production, activity of NAPDH oxidase and levels of 4-HNE and MDA in DOX-treated hearts. Moreover, these oxidative alterations were enhanced after FL treatment *in vivo* and *in vitro*. The profound involvement of TLR5 in oxidative stress reported by our study is consistent with previous studies 47,48. Moreover, our results demonstrated that FL increased the production of superoxide through a TLR5-dependent but MyD88-independent mechanism, implying there was another molecule that connected TLR5 with superoxide that was involved in FL-induced toxicity. Here, we found that TLR5 depletion reduced NOX2 expression and FL increased NOX2 expression. These findings are in agreement with a previous report that NOX2 was responsible for the production of superoxide 49. Next, we tested the hypothesis that NOX2 is responsible for the role of TLR5 in DOX-related cardiac injury. We found that the toxic effects of FL in DOX-treated hearts and cells were abolished in NOX2 deficient mice and cells, suggesting that the effects of TLR5 are mediated by NOX2.

Next, we determined how TLR5 activates NOX2. The data in our study indicated that there was no direct interaction between TLR5 and NOX2, thus we sought a signaling pathway to link TLR5 and NOX2. Activation of NOX2 requires membrane translocation of cytoplasmic subunits p47phox, p67phox and Rac to a membrane-bound heterodimer cytochrome comprised of gp91phox and p22phox 50. A canonical mechanism of NOX2 activation requires PLCγ1-dependent PKC activation, leading to phosphorylation of p47phox and membrane translocation of cytoplasmic subunits p47phox 33,51. Syk phosphorylates PLCγ1 and plays a key role in the production of ROS 34,35. Here, for the first time, we demonstrated that there is a direct interaction between Syk and TLR5. Syk phosphorylation in response to DOX was largely prevented by TLR5 deficiency. Using Syk inhibition, we found that in turn, Syk was required for FL-induced PLCγ1 activation and superoxide generation. In line with this finding, we further observed that FL promoted DOX-induced membrane translocation of PKCα. Thus, we found that Syk and sequential activation of downstream targets was responsible for FL/TLR5-mediated NOX2 activation and superoxide generation.

p38, a member of the mitogen-activated protein kinase family, is activated by growth and oxidative stress signals 52. Mice with dominant-negative p38α exhibited improved cardiac function in response to DOX injection 53. Here, the data in our study demonstrated that DOX-induced activation of p38 was prevented by TLR5 deficiency but enhanced by FL treatment. A previous study found that ROS activate the p38 signaling pathway 54. Consistent with this, we also found that p38 activation after FL treatment was regulated by NOX2-dependent superoxide production. p38 is a mediator of DOX-induced apoptosis, and inhibition of p38 suppresses DOX-induced cardiomyocyte apoptosis 55. Consistent with this finding, we found that inhibition of p38 prevented the decreased viability in FL+DOX-treated cells, suggesting that FL/TLR5 impairs cardiomyocyte viability via activation of p38.

Our study also has limitations. The toxic effects observed upon FL injection cannot be simply attributed to a cardiac restricted action. These effects are a part of the systemic reaction to this bacterial protein 18. In the present study, we used a single injection strategy to mimic DOX-related acute cardiac injury. A more clinically relevant, chronic model was not included in our study. In addition, we did not examine the effect of TLR5 depletion on antineoplastic action of DOX.

Collectively, our study found that TLR5 deficiency attenuated DOX-induced cardiomyopathy and improved cardiac function. NOX2 activation and superoxide generation, caused by direct interaction between TLR5 and Syk, and sequential p38 activation were responsible for the role of TLR5 in DOX-induced cardiac injury.

## Supplementary Material

Supplementary figures and tables.
