# Supplementary material for: Toll-like receptor 5 deficiency diminishes doxorubicin-induced acute cardiotoxicity in mice
Source: Theranostics. 2020 Sep 2;10(24):11013–25. doi: 10.7150/thno.47516 (PMC7532690; doi:10.7150/thno.47516)
Supplement: Supplementary file 1 — Supplementary figures and tables. [file thnov10p11013s1.pdf]

Supplement

Toll-like receptor 5 deficiency diminishes doxorubicin-induced acute cardiotoxicity in mice

Running title: TLR5 deficiency diminishes acute cardiotoxicity

Zhen-Guo Ma[1,2*], Chun-Yan Kong[1,2], Hai-Ming Wu[1,2], Peng Song[1,2], Xin Zhang[1,2], Yu-Pei Yuan[1,2], Wei Deng[1,2], Qi-Zhu Tang[1,2*]

[1]Department of Cardiology, Renmin Hospital of Wuhan University, Wuhan 430060, RP China

[2]Hubei Key Laboratory of Metabolic and Chronic Diseases, Wuhan 430060, RP China

Zhen-Guo Ma and Chun-Yan Kong contributed equally to this study

[*]Corresponding author:

Zhen-Guo Ma,
Department of Cardiology,
Renmin Hospital of Wuhan University,
Hubei Key Laboratory of Metabolic and Chronic Diseases,
Wuhan University at Jiefang Road 238, Wuhan 430060, RP China
Tel.: +86 27 88073385; Fax: +86 27 88042292. E-mail: zhengma@whu.edu.cn

Qi-Zhu Tang,
Department of Cardiology,
Renmin Hospital of Wuhan University,
Hubei Key Laboratory of Metabolic and Chronic Diseases,
Wuhan University at Jiefang Road 238, Wuhan 430060, RP China
Tel.: +86 27 88073385; Fax: +86 27 88042292. E-mail: qztang@whu.edu.cn

Figure S1

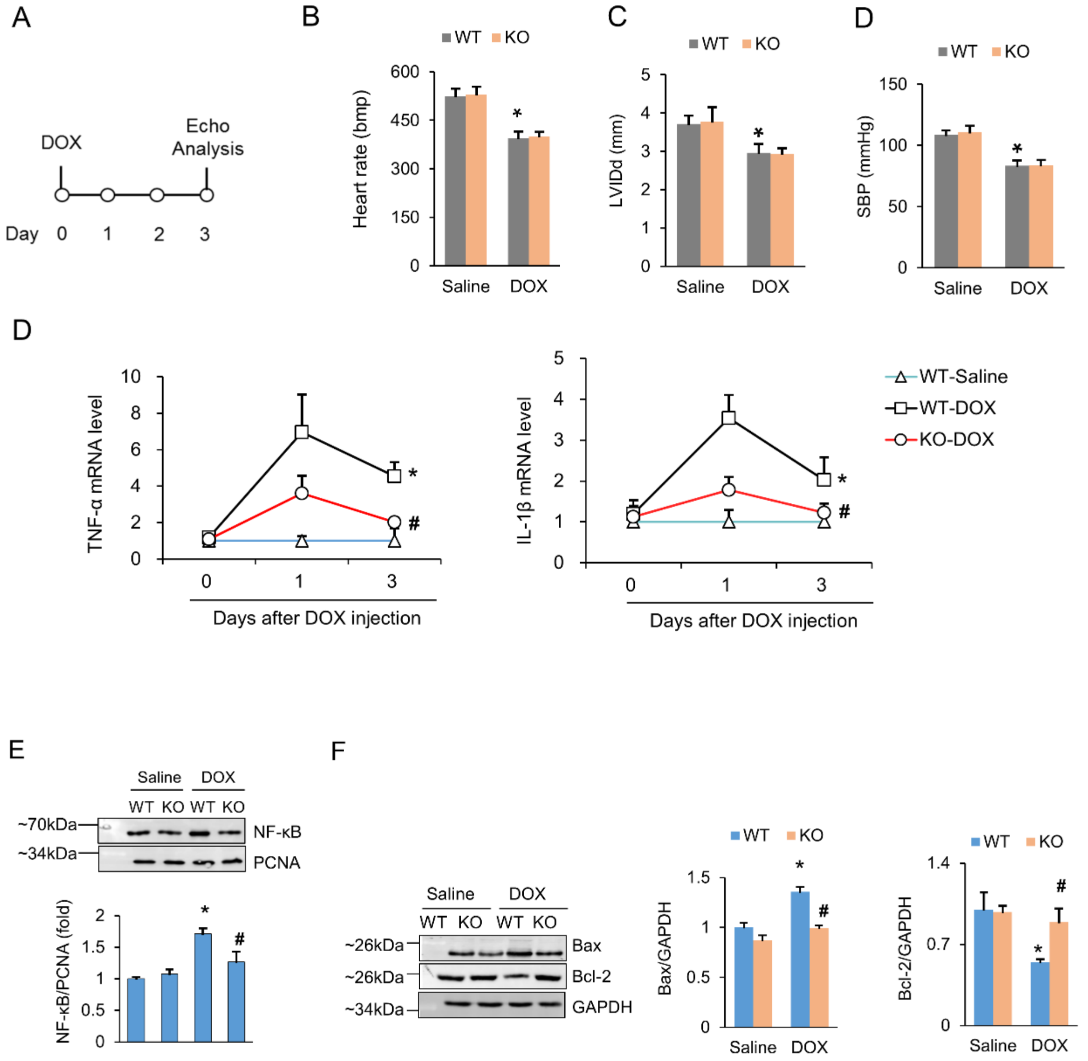

Figure S1. TLR5 deficiency attenuates DOX-induced inflammatory response and apoptosis without affecting cardiac function in mice. (A) Experimental protocol. (B-D) The alterations of heart rate, LVIDd and SBP after DOX injection in mice (n=6). (E) The mRNA levels of inflammatory factors in mice (n=6). (F) Nuclear translocation of NF-κB (n=6). (G) The protein expressions of pro/anti-apoptotic gene (n=6). **P*<0.05 versus WT Saline; #*P*<0.05 versus WT DOX group.

Figure S2

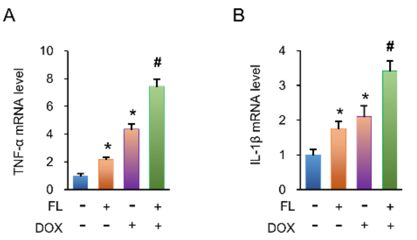

Figure S2. FL exacerbates DOX-induced inflammation in mice. (A) TNF-α mRNA level (n=6). (B) IL-1β mRNA level (n=6). **P*<0.05 versus the matched control. vehicle; #*P*<0.05 versus DOX group.

Figure S3

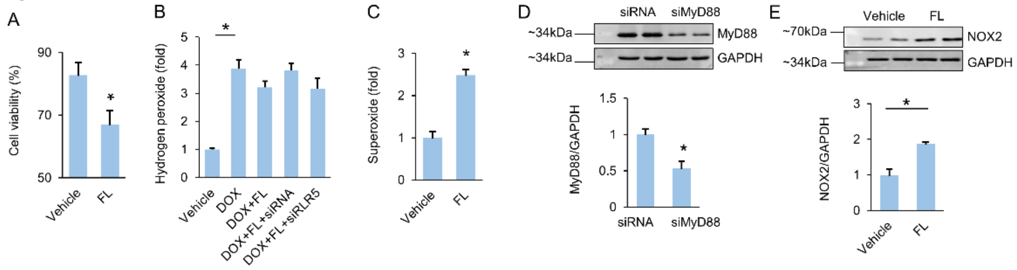

Figure S3. The production of ROS induced by DOX is TLR5 dependent. (A) Cell viability after FL treatment (n=6). (B-C) The production of hydrogen peroxide and superoxide (n=6). (D) The protein expression of MyD88 (n=6). (E) The protein expression of NOX2 after FL treatment (n=6). *P<0.05 versus the matched control.

Figure S4

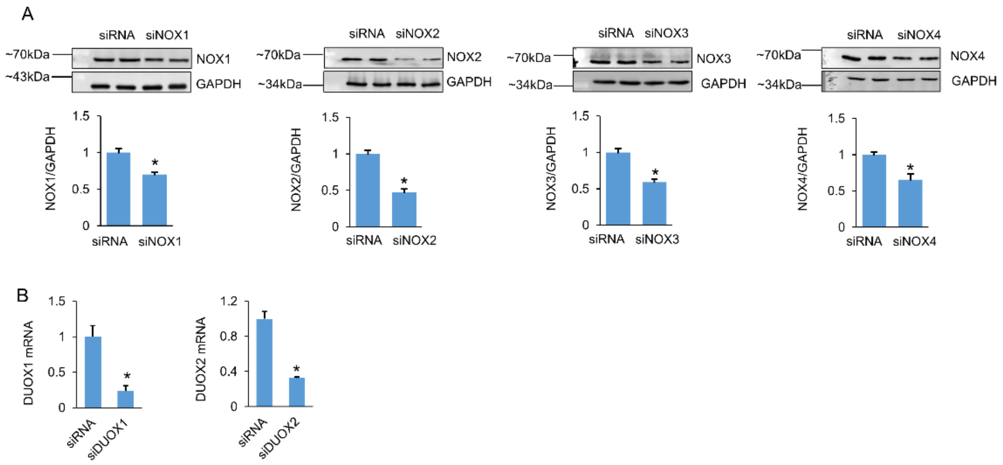

Figure S4. The deficiency of NOX isoenzymes. (A) The protein expression of NOX isoenzymes (n=6). (B) The mRNA levels of DUOX1 and DUOX2 (n=6). *P<0.05 versus the matched control.

Figure S5

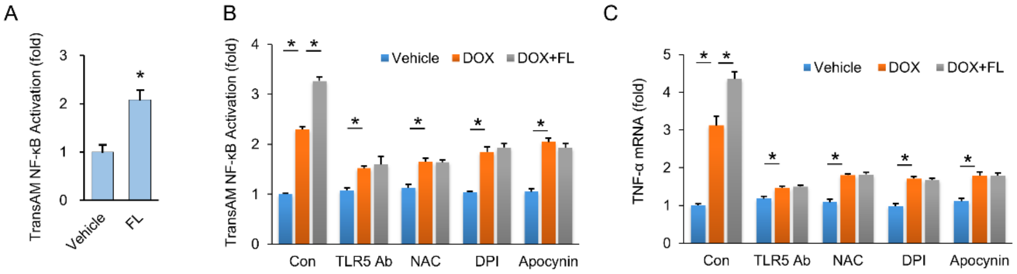

Figure S5. TLR5 promotes DOX-induced inflammation via ROS production. (A-B) NF-κB activation (n=6). (C) TNF-α mRNA level (n=6). *P<0.05 versus matched control. The data are expressed as the mean ± SEM from three independent experiments.

Figure S6

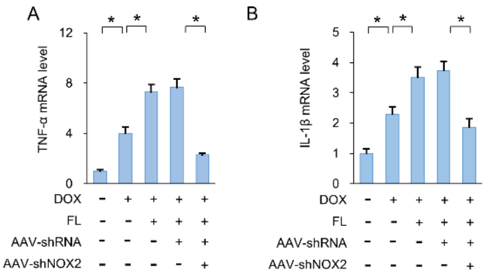

Figure S6. NOX2 deficiency suppresses the inflammatory response caused by DOX in mice. (A-B) The mRNA levels of inflammatory factors in mice (n=6). **P*<0.05 versus matched control.

Figure S7

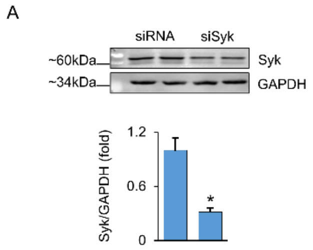

Figure S7. The protein expression of Syk in vitro (n=6). **P*<0.05 versus matched control.

Figure S8

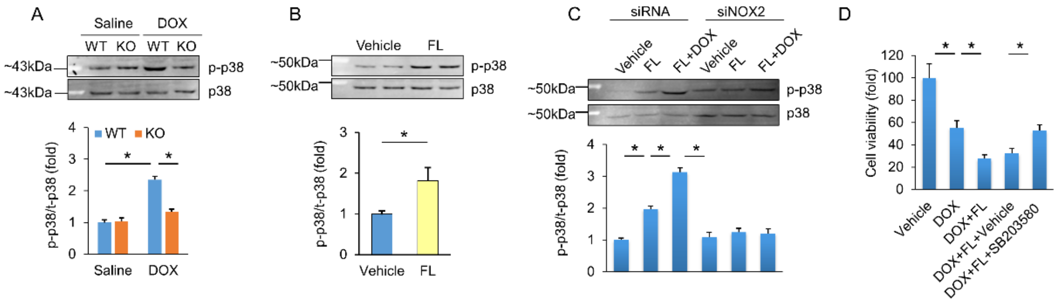

Figure S8. p38 inhibition blockes the toxic effects of FL on cell viability in vitro. (A-C) The protein expressions of p38 (n=6). (D) Cell viability after p38 inhibition (n=6). **P*<0.05 versus matched control.

Figure S9

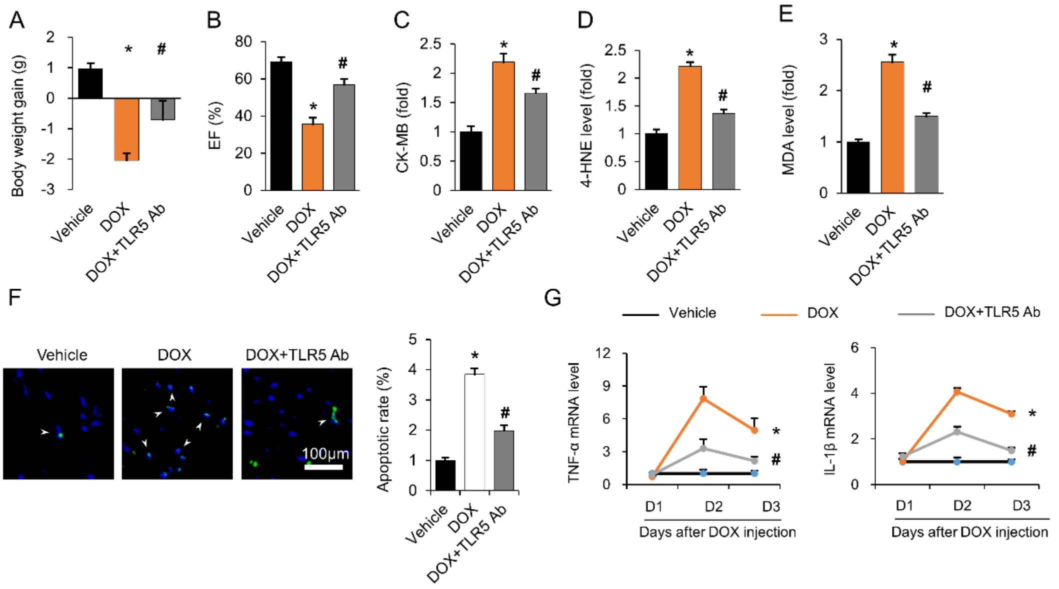

Figure S9. TLR5 neutralizing antibody abolished the cardiac damage caused by DOX in mice. (A) Body weight change (n=10). (B) Ejection fraction (n=8). (C) CK-MB in DOX-treated mice (n=6). (D-E) 4-HNE and MDA production (n=6). (F) TUNEL staining (n=6). (G) The mRNA levels of inflammatory factors (n=6). *P<0.05 versus matched control; #P<0.05 versus DOX group.
